# Seroprevalence of hepatitis C virus infection in patients with type 2 diabetes mellitus is associated with increased age in sub-Saharan Africa: Results from a cross-sectional comparative analysis

**DOI:** 10.3389/fgstr.2023.1063590

**Published:** 2023-01-23

**Authors:** Charly Feutseu, Mathurin Pierre Kowo, Anne Ongmeb Boli, Jean Claude Katte, Magellan Guewo-Fokeng, Sylvain Zemsi, Mesmin Yefou Dehayem, Simeon Pierre Choukem, Eugene Sobngwi

**Affiliations:** ^1^ Laboratory for Molecular Medicine and Metabolism, Biotechnology Center, University of Yaoundé 1, Yaoundé, Cameroon; ^2^ Hepato-Gastroenterology Unit, Yaounde Teaching Hospital, Yaoundé, Cameroon; ^3^ National Obesity Center and Endocrinology and Metabolic Diseases Unit, Yaoundé Central Hospital, Yaoundé, Cameroon; ^4^ Recherche Sante et Developpement (RSD) Institute, Yaoundé, Cameroon; ^5^ Faculty of Medicine and Biomedical Sciences, University of Yaoundé 1, Yaoundé, Cameroon; ^6^ Faculty of Medicine and Pharmaceutical Sciences, University of Dschang, Dschang, Cameroon

**Keywords:** HCV infection, type 2 diabetes mellitus, seroprevalence, sub-Saharan Africa, age-dependent association

## Abstract

**Background:**

Several epidemiological studies have established the association between hepatitis C virus (HCV) infection and type 2 diabetes mellitus (T2DM). However, the determinants and reasons for the high prevalence of HCV infection in people with T2DM are not well understood especially in sub-Saharan African populations. In this study, we aimed to assess determinants of the association between HCV infection and T2DM.

**Methods:**

We performed a cross-sectional study amongst 442 T2DM patients recruited from an out-patient adult diabetes clinic in a tertiary hospital and 442 non-diabetic controls recruited from the general population. Serological testing for HCV antibody was performed using standard ELISA technique. Anti-HCV antibody prevalence was reported by age group in participants with diabetes and the non-diabetic controls. Logistic regression was used to examine for factors associated with the HCV infection in patients with diabetes.

**Results:**

We reported an overall HCV prevalence of 11.5% [95% CI: 9.4-13.6] irrespective of diabetes status in this study. The seroprevalence of HCV infection in diabetics patients was 17.6% [95% CI: 14.0-21.2] compared to 5.5% [95% CI: 3.4-7.6] in non-diabetics (p< 0.001). We did not find a significant HCV seropositivity difference in diabetic patients with common risk factors of HCV infection. When investigating the HCV seroprevalence by age group in diabetic and non-diabetic patients, no case of HCV infection was found in patients less than 30 years old while the highest HCV seropositivity was reported in patients older than 60 years (36.7% T2DM and 11.1% for non-diabetics) followed by the patients belonging to 50-59 years age group (16% in T2DM and 5.8% in non-diabetics) and those in 40-49 years age group (4.4% in diabetic, 0.8% in non-diabetic). To support this finding, in a multivariate logistic regression, only diabetic patients belonging to age group > 65 years had a significant risk (OR: 16.7 [95% CI: 1.7-160.0]) to acquire HCV infection.

**Conclusion:**

The seroprevalence of HCV infection is higher among T2DM adult patients than in non-diabetic patients, and is associated with increased age. This age-dependent association may suggest a generational exposure that may no longer exist overtime.

## 1 Introduction

Hepatitis C virus (HCV) infection and diabetes mellitus (DM) are two common disorders that cause devastating long-term complications in a significant number of patients worldwide. A recent data report by the World Health Organization estimated that about 71 million people worldwide were living with chronic HCV infection which represents about 1% of the global population (1). This disease is a frequent cause of cirrhosis and hepatocellular carcinoma and was responsible for about 580,000 deaths across the world in 2018 (2). Meanwhile, data reported by the International Diabetes Federation (IDF) in 2021 showed that about 537 million people have DM worldwide with a global prevalence of approximately 10.5% (3). Both diseases present a large healthcare burden in developed countries as well as in developing countries.

In sub-Saharan Africa, the estimated prevalence of HCV and DM is about 2.9% and 4.5% respectively (3, 4). Type 2 diabetes mellitus (T2DM) represents about 95% of the entire population with DM (5). This disease is a multifactorial condition that results from an interaction between genetic and environmental factors (6). However, the underlying mechanism is not well understood. The likely etiology is a combination of factors, including age, genetic inheritance and environmental factors such as lifestyle and viral infections (7). Concerning viral infections, there are increasing number of reports on the diabetogenic role of HCV (8–10). It has been observed that cirrhotic patients infected with HCV may present with T2DM more often than patients with cirrhosis of other etiologies (11). This preliminary observation was confirmed thereafter by other epidemiological studies performed on different ethnic groups. The link between HCV and T2DM has been described further in the literature. A meta-analysis of 34 published studies with 300,000 HCV infected patients found a 1.7 fold increase in risk of DM compared to HCV negative controls (12). Another study showed that type 2 diabetic patients were shown to have approximately a 3 fold increased risk to acquire HCV infection when compared to non-diabetic controls (13). Moreover, a high prevalence of HCV infection was reported in type 2 diabetic patients in a meta-analysis performed by Fabiani and colleagues in 2018 (14). The high prevalence of HCV infection in diabetic patients could be related to the high transmission rate of hepatitis C virus from the 1920 to 1980 period, due to the use of unsterile injection equipment for mass treatment of the general population and parenteral infection on the occasion of mass medical campaigns (15, 16). Therefore, we undertook this study in order to assess determinants of the association between HCV infection and type 2 diabetes mellitus in a Sub-Saharan African population.

## 2 Methods

### 2.1 Study design, setting and population

This was a cross-sectional study that took place from May 2015 to June 2017 at the National Obesity Centre (NOC), Yaoundé Central Hospital, a tertiary hospital in Yaoundé, Cameroon. T2DM patients attending the out-patient department of the National Obesity Centre for their routine visit were consecutively recruited during their routine diabetes clinic consultations. Non-diabetic individuals were recruited from the general population based on similar residential catchment areas to the recruited people with diabetes.

### 2.2 Data collection

Socio-demographic and clinical characteristics were recorded using a standard pre-tested and validated electronic data collection form (questionnaire) using a hand-held tablet. Relevant clinical data on the history of previous exposure to possible risk factors for HCV infection (intravenous drug use, tattooing/scarification, blood transfusion, surgical or dental treatment, and alcohol consumption) were collected. Serological testing for hepatitis C antibody was performed using a third-generation Enzyme Immunosorbent Assay (ELISA) (Rapid Labs Limited, United Kingdom) according to the manufacturer’s instructions. HbA1c was also measured in patients with T2DM. Diabetes control was defined as good (HbA1c< 7%), moderate (HbA1c 7-8%) or poor (HbA1c> 8%) control. Body mass index (BMI) was calculated as the ratio of the weight (kg) to the height (m)^2^ then classified as underweight (<18.5 kg/m^2^), normal (18.5 – 24.9 kg/m^2^), overweight (25 – 29.9 kg/m^2^) and obese (> 30 kg/m2). A total of 442 type 2 diabetic patients and 442 non-diabetic controls were enrolled in this study.

### 2.3 Ethical considerations

Ethical clearance was obtained from the National Ethics Committee for Human Health Research at the Ministry of Public Health, Cameroon (N°2014/04/437/I/CNERSH/SP). Before enrollment in the study, each participant provided a signed written informed consent.

### 2.4 Statistical analysis

Data were analyzed using the Statistical Package for Social Science (SPSS) version 23.0 (SPSS lnc, Chicago, IL, USA). The results were presented as means ± standard deviation. Categorical variables were presented in proportions. Comparison between groups was performed using a two-sided student’s t-test for continuous variables and Chi-squared test for categorical variables. Logistic regression was used to assess for factors associated with HCV infection in patients with type 2 diabetes mellitus. The level of significance was set at 0.05 for all analyses.

## 3 Results

### 3.1 Socio-demographic and clinical characteristics

Overall, we included 884 patients (442 diabetic patients and 442 non-diabetic controls) in this study. **Table 1** shows the socio-demographic and clinical characteristics of the patients with type 2 diabetes mellitus and non-diabetic controls. Overall, the majority of patients were married (70.4%), female (69.8%), and attended secondary school (52.3%). The mean BMI was significantly higher in non-diabetic controls compared to diabetic patients (29.91 ± 6.25 Kg/m^2^ vs 31,1 ± 5.66 Kg/m^2^, P = 0.004) while waist circumference (98.06 ± 13.54 cm vs 98.79 ± 12.73 cm, P = 0.76) and diastolic blood pressure (DBP) (77 ± 11 mmHg vs 77 ± 12 mmHg, P = 0.72) were comparable in both groups.

**Table 1:** General Sociodemographic and clinical characteristics of diabetic and non-diabetic patients.

Characteristics	Overall N = 884	Diabetic patients N = 442	Non-diabetic controls N = 442
Age (years)*	52.9 ± 10.5	53.1 ± 10.3	52.8 ± 10.7
Age group n (%)			
> 65	20 (13.6)	60 (13.6)	60 (13.6)
40-65	686 (77.6)	343 (77.6)	343 (77.6)
< 40	78 (8.8)	39 (8.8)	39 (8.8)
Gender n (%)			
Male	267(30.2)	149(33.7)	118(26.7)
Marital status n (%)			
Married/cohabiting	622(70.4)	307(69.5)	315(71.3)
Divorced	29(3.3)	17(3.8)	12(2.7)
Widowed	158(17.9)	73(16.5)	85(19.2)
Single	75(8.5)	45(10.2)	30(6.8)
Education level n (%)			
Primary	266(33.3)	126(29.9)	140(37.0)
Secondary	418(52.3)	229(54.4)	189(50.0)
Higher	115(14.4)	66(15.7)	49(13.0)
BMI (Kg/m^2^)*	30.4 ± 5.9	29.9 ± 6.2	31.1 ± 5.6
WC (cm)*	98.9 ± 13.1	98.0 ± 13.5	98.7 ± 12.7
DBP(mmHg)*	76 ± 11	76 ± 11	76 ± 12
SBP(mmHg)*	123 ± 18	125 ± 19	122 ± 18

* mean ± Standard Deviation, WC, Waist circumference; BMI, Body mass index; SBP, Systolic Blood Pressure; DBP, Diastolic Blood Pressure.

### 3.2 History of exposure to risk factors of HCV infection

**Table 2** shows an analysis of known risk factors for HCV infection. History of blood transfusion (13.7% vs 11.8%, P = 0.47), tattooing or scarification (36.1% vs 38.6%, P = 0.48), injected drug (0.5% vs 0%, P = 0.49) and surgical or dental treatment (62.2% vs 61.9%, P = 0.94) were comparable in diabetic patients and non-diabetic controls. History of smoking were reported in a significantly high number of diabetic patients compared to non-diabetic controls (13.3% vs 7.9, P= 0.03).

**Table 2:** HCV seroprevalence and Analysis of known risk factors of HCV infection in patients with type 2 diabetes and non-diabetic controls.

Characteristics	Overall N = 884	Diabetic patients N = 442	Non-diabetic controls N = 442	P-value
HCV antibodies** n (%)				< 0.001
Positive	99 (11.5)	75 (17.6)	24 (5.5)	
**Blood transfusion n (%)**				0.47
Yes	112 **(**12.8)	60 (13.7)	52 (11.8)	
**Piercing n (%)**				0.03
Yes	310 (35.2)	139 (31.7)	171 (38.8)	
**Tattoo or cuts n (%)**				0.48
Yes	311 (37.3)	151(36.1)	160 (38.6)	
**Injected drug n (%)**				0.49
Yes	2 (0.2)	2(0.5)	0 (0)	
**Surgical/dental treatment n (%)**				0.94
Yes	546 (62.0)	273 (62.2)	273 (61.9)	
**Smoking n (%)**				0.01
Yes	94 (10.6)	59 (13.3)	35 (7.9)	
**Alcohol consumption n (%)**				< 0.001
Yes	501 (84.3)	260 (77.8)	241 (92.7)	

** Anti-HCV serology was available for 863 patients: 427 diabetic patients and 436 non-diabetic controls.

### 3.3 Prevalence of anti-HCV antibody

**Figure 1:**
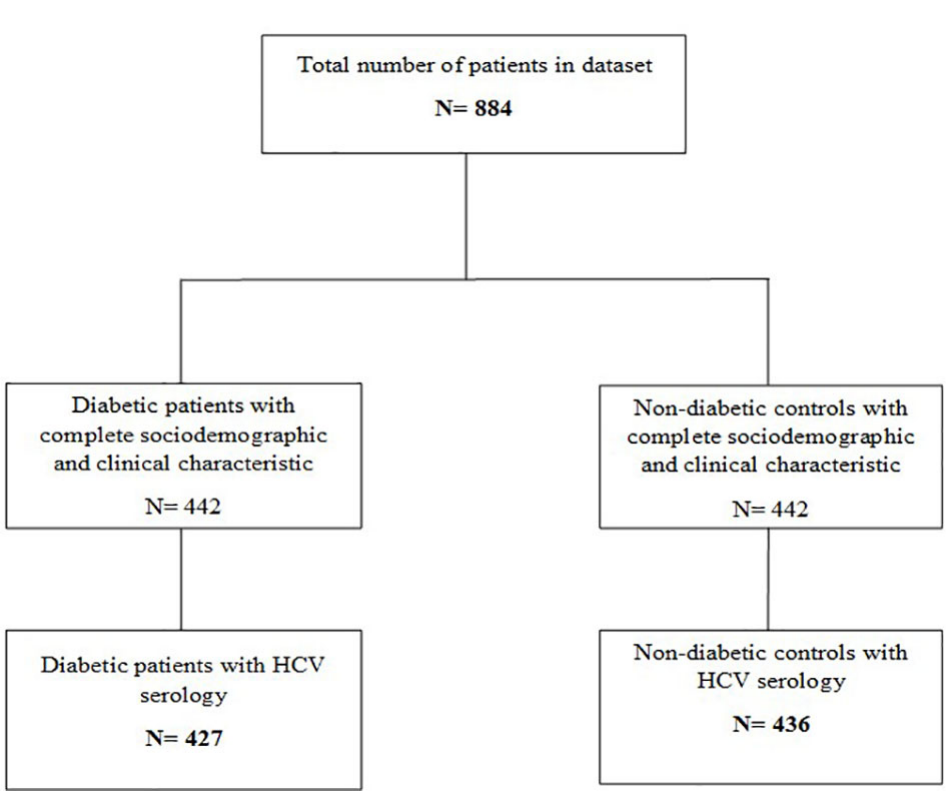
Flow diagram of the participants.

Anti-HCV serology was available for 863 study participants (427 diabetic patients and 436 non-diabetic controls) (**Figure 1**). Overall, evidence of HCV infection was found in 99 participants, giving an overall prevalence irrespective of diabetes status of 11.5% [95% CI: 9.4-13.6]. When comparing diabetic and non-diabetic patients, we found serological markers of HCV infection in 75 (17.6% [95% CI: 14.0-21.2]) diabetic patients compared to 24 (5.5% [95% CI: 3.4-7.6]) non-diabetic controls. Indeed, the anti-HCV antibody prevalence was significantly higher in diabetic patients compared to non-diabetic controls (p< 0.001) (**Table 2**).

When considering HCV seroprevalence according to clinical characteristics and history of exposure to HCV infection in diabetic patients, we found that a larger proportion of HCV seropositivity (39.0%) was observed in patients who belonged to the age group > 65 years. Patients having diabetes mellitus for more than 10 years had higher HCV seropositivity (30.3%). Meanwhile, patients with a history of alcohol consumption and those with good glycemic control had high HCV seropositivity. Concerning the history of blood transfusion, tattooing or scarification, injected drug, piercing, gender, marital status, education level, and smoking, we did not find a significant HCV seropositivity difference in diabetic patients with these medical histories compared to those without (**Table 3**).

**Table 3:** Seroprevalence of HCV infection in type 2 diabetic patients according to clinical characteristics and history of exposure to HCV infection.

Characteristics	Anti-HCV(-) N = 352	Anti-HCV(+) N = 75	P-value
Age (Years)*	51.8 (9.7)	60.1 (9.9)	< 0.001
BMI (Kg/m^2^)*	30.1 (6.2)	28.9 (6.5)	0.13
HbA1c (%)*	7.6 (2.2)	7.0 (1.9)	0.04
Duration of diabetes (years)*	5.0 (5.9)	9.0 (8.4)	< 0.001
**Age group n (%)**			< 0.001
> 65	36(61.0)	23(39.0)	
40-65	282(85.2)	49(14.8)	
< 40	34(91.9)	3(8.1)	
**Gender n (%)**			0.97
Male	118 (82.5)	25 (17.5)	
**Education level n (%)**			0.07
Primary	96(76.8)	29(23.2)	
Secondary	185(84.5)	34(15.5)	
Higher	56(88.9)	7(11.1)	
**Marital status n (%)**			0.69
Married/Cohabiting	248(83.8)	48(16.2)	
Divorced	14(82.4)	3(17.6)	
Widowed	56(80.0)	14(20.0)	
Single	34(77.3)	10(22.7)	
**Alcohol consumption n (%)**			0.02
Yes	214 (85.3)	37(14.7)	
**Smoking n (%)**			0.852
Yes	48 (84.2)	9(15.8)	
**BMI categories n (%)**			0.106
< 25	81(75.7)	26(24.3)	
> 30	136(85.0)	24(15.0)	
25-30	135(84.4)	25(15.6)	
**Diabetes control n (%)**			0.03
Good	177(78.7)	48(21.3)	
Poor	119(89.5)	14(10.5)	
Moderate	46(79.3)	12(20.7)	
**Duration of diabetes categories**			< 0.001
<5 years	210(87.1)	31(12.9)	
5-10 years	64(84.2)	12(15.8)	
>10 years	69(69.7)	30(30.3)	
**Insulin user n (%)**			0.16
Yes	111 (79.3)	29 (20.7)	
**Blood transfusion n (%)**			0.19
Yes	45 (76.3)	14(23.7)	
**Tatoo/Cuts n (%)**			0.22
Yes	124 (85.5)	21 (14.5)	
**Surgical/dental treatment n (%)**			0.69
Yes	221 (83.4)	44 (16.6)	
**Piercing n (%)**			0.28
Yes	108(79.4)	28(20.6)	
**Injected drug n (%)**			0.52
Yes	2 (100.0)	0(0)	

* mean ± Standard Deviation.

### 3.4 Prevalence of anti-HCV by age group in diabetic patients and non-diabetic controls

**Figure 2:**
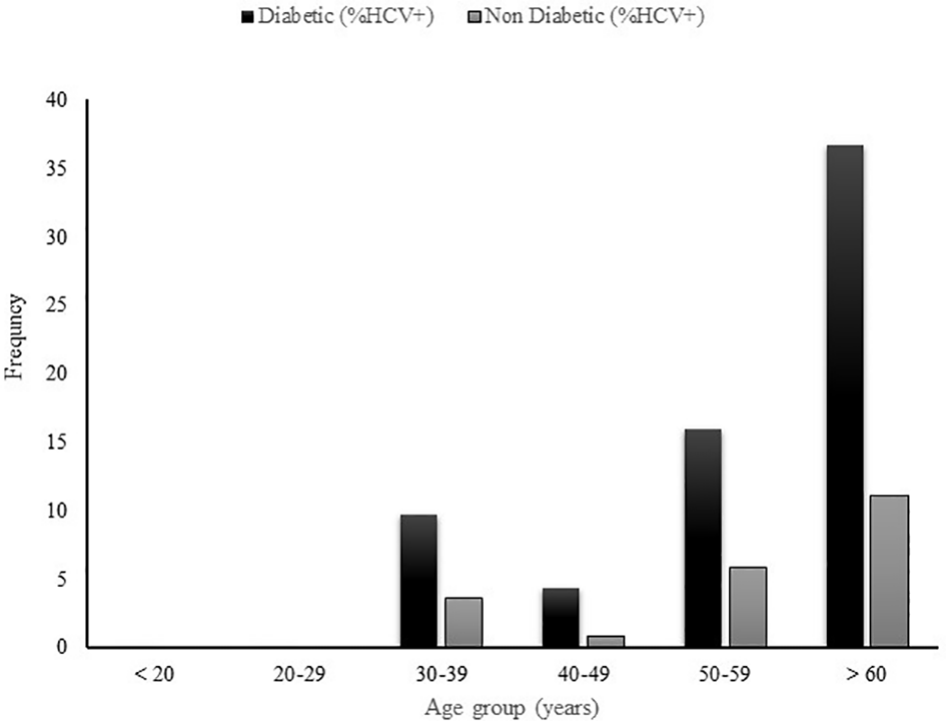
Prevalence of HCV infection in diabetic and non-diabetic patients by age group.

In order to further assess the age-related HCV prevalence in diabetic patients, the diabetic and non-diabetic patients were subdivided into several age groups as depicted in **Figure 2**. The prevalence of HCV infection was assessed thereafter in each age group. In both groups, no case of HCV infection was found in patients less than 30 years. The highest HCV seropositivity was reported in patients older than 60 years (36.7%(40/109) in diabetic patients and (11.1%(12/108) for their non-diabetic peers) followed by the patients belonging to 50-59 years age group (16%(27/169) in diabetic patients and 5.8%(10/172) in non-diabetic controls) and those in 40-49 years age group (4.4%(5/113) in diabetic patients, 0.8%(1/118) in non-diabetic controls).

To support this finding, in a multivariate logistic regression, only diabetic patients belonging to age group > 65 years had a significant risk (OR: 16.7 [95% CI: 1.7-160.0]) to acquire HCV infection. None other variables included in the model were associated with the increase risk of HCV infection in diabetic patients (**Table 4**).

**Table 4:** Logistic regression examing the determining factors for the HCV infection and type 2 diabetes mellitus.

Characteristics	Overall N = 324	Anti-HCV(+) N = 54	Adjusted OR (95% CI)	P-value
Age group n(%)				
> 65	39 (12.0)	16 (41.0)	16.7(1.7-160.0)	0.02
40-65	254 (78.4)	37 (14.6)	5.1(0.6-42.7)	0.14
< 40	31 (9.6)	1 (3.2)	1	
Gender n (%)				
Male	119 (36.7)	23 (19.3)	1.9(0.7-5.0)	0.20
Female	205 (63.3)	31 (15.1)	1	
Marital status				
Married/Cohabiting	224 (64.1)	35 (15.6)	0.3(0.1-1.0)	0.05
Divorced	12 (3.7)	2 (16.7)	0.5(0.07-3.4)	0.48
Widowed	51 (15.7)	10 (19.6)	0.4(0.1-1.3)	0.11
Single	37 (11.4)	7 (18.9)	1	
Education level n(%)				
Primary	98 (30.2)	21 (21.4)	1.6(0.5-5.2)	0.40
Secondary	177 (54.6)	26 (14.7)	1.0(0.4-3.0)	0.93
Higher	49 (15.1)	7 (14.3)	1	
BMI categories n(%)				
> 30	126 (38.9)	17 (13.5)	0.7(0.3-1.7)	0.42
25-30	124 (38.3)	21 (16.9)	0.6(0.3-1.5)	0.31
< 25	74 (22.8)	16 (21.6)	1	
Diabetes control n(%)				
Good	174 (53.7)	35 (20.1)	1.2(0.5-3.2)	0.71
Poor	103 (31.8)	9 (8.7)	0.4(0.1-1.4)	0.18
Moderate	47 (14.5)	10 (21.3)	1	
Duration of diabetes n(%)				
> 10 yrs	82 (25.3)	24 (29.3)	2.4(1.1-5.3)	0.03
5-10 yrs	60 (18.5)	8 (13.3)	0.9(0.3-2.3)	0.79
< 5 yrs	182 (40.7	22 (12.1)	1	
Insulin user n(%)				
Yes	122 (37.7)	24 (19.7)	1.1(0.6-2.3)	0.73
No	202 (62.3)	30 (14.9)	1	
Smoking				
Yes	46 (14.2)	7 (15.2)	0.8(0.3-2.3)	0.63
No	278 (85.8)	47 (16.9)	1	
Blood transfusion n(%)				
Yes	46 (14.2)	11 (23.9)	2.5(1.0-5.9)	0.04
No	278 (85.8)	43 (15.5)	1	
Tatoo/Cuts n(%)				
Yes	114 (35.2)	14 (12.3)	0.6(0.3-1.4)	0.26
No	210 (64.8)	40 (19.0)	1	
Piercing n(%)				
Yes	85 (26.3	14 (16.5)	1.3(0.6-3.0)	0.56
No	239 (73.7)	40 (16.7)	1	
Surgical treatment n (%)				
Yes	198 (61.1)	31 (15.7)	0.7(0.4-1.5)	0.38
No	123 (38.9)	23 (18.3)	1	

## 4 Discussion

The present study aimed to assess determinants of the association between HCV infection and type 2 diabetes mellitus in a sub-Saharan African population. We found a high prevalence of HCV infection (17.6%) in Cameroonian patients with type 2 diabetes mellitus compared to non-diabetic controls (5.5%). Furthermore, almost all the cases of HCV seropositivity were reported in patients older than 40 years old in both diabetic patients and non-diabetic controls, suggesting that the association between HCV infection and T2DM is mostly observed in patients born before 1980s.

The association between T2DM and HCV infection was reported previously by several epidemiological studies performed on different ethnic groups across the world (9, 17). For the first time, we reported in this study, a high prevalence of HCV infection among type 2 diabetic patients compared with non-diabetic controls in a Cameroonian population. This prevalence is much higher than the prevalence of 1.9% reported in Saudi Arabia (18), 11% in Nigeria (19) and 2.4% in Brazil (20). The higher prevalence of HCV infection in diabetic patients reported in our study suggests that the association between the two diseases may present some characteristics in our setting. This can be related to HCV genotype, ethnicity or the high prevalence of HCV infection in the general population. In fact, Sub-Saharan Africa, with a prevalence of about 3% (4), bears the highest burden of HCV infection. Furthermore, with a prevalence of about 6%, Cameroon is classified among the countries with highest prevalence of HCV infection in the world (21). This high seropositivity rate of HCV infection in Sub-Saharan Africa in general and particularly in Cameroon, can partly explain the high prevalence of HCV infection in diabetic patients reported in this study.

It was previously shown that the higher risk of HCV infection in type 2 diabetic patients may be related to frequent parenteral exposure (self-monitoring of blood glucose, insulin injection, blood transfusion) (22, 23). Moreover, blood transfusion, mother-to-child transmission, non-sterile medical procedures and traditional practices (scarification, tattoo, and circumcision) were considered as common routes of HCV infection in Sub-Saharan Africa, despite the absence of confirmatory studies (16, 24). However, in this study, neither insulin injection nor the history of common risk factors of HCV infection (blood transfusion, surgical treatment, tattooing or scarification and drug injection) were significantly associated with HCV seropositivity in type 2 diabetic patients. Similar results were also reported by Kombi et al. in a Democratic Republic of Congo population (25), Jadon et al. in a Pakistan population (26) and Chen et al. in Taiwan population (23). Indeed, the high prevalence of HCV in diabetic patients reported in this study cannot be explained by traditional risk factors of HCV infection in our setting.

In order to further investigate the factors related to the high prevalence of HCV infection in diabetic patients reported in this study, we assessed the HCV prevalence by age group in diabetic patients and non-diabetic controls. We found that almost all cases of HCV seropositivity were reported in patients older than 40 years in both diabetic and non-diabetic patients. Consistent with this finding, in multivariate logistic regression, only type 2 diabetic patients belonging to age group > 65 years had a higher risk to acquire HCV infection. This could suggest a generational effect concerning the association between HCV infection and T2DM. Therefore, we can assume with caution that this association is mostly observed in patients born before 1980s and may no longer exist overtime. The high circulation of hepatitis C virus occurred in the world and particularly in SSA during 1920 to 1980 period due to the use of unsterile injection equipment for mass treatment of the general population and parenteral injection on the occasion of mass medical campaigns which consisted of repeated injections, often using non-sterile materials and serial arm to arm procedures (15, 16). Similarly, multi injection treatment for trypanosomiasis in Central Africa facilitated a widespread transmission of HCV from 1920 to 1940 (27). For example, between 1920 and 1960, the seroprevalence of HCV infection significantly increased in Cameroon, coinciding with mass campaigns of vaccination and treatment for trypanosomiasis (24). The continuous exposure to infection during a lifetime may explain the increasing prevalence of HCV infection with age (16). Thus, our findings revealed that comorbid HCV infection and type 2 diabetes mellitus is strongly associated with age. As strongly suggested, this could be also related to a compromised immune system that is associated with an increase in age. Previous findings shown that people with diabetes are predisposed to infections and that infection complicates the control of the diabetes. In general, viral infections are more frequent in diabetic patients, which potentially increase their morbid-mortality. The greater frequency of infections in diabetic patients could be related to the hyperglycemic environment that favors immune dysfunction such as the damage to the neutrophil function, depression of the antioxidant system, impairment of cytokines production and humoral immunity (28–30). However, we did not assess the immunity factors of patients included in this study. we can not assume with confidence that the age-related HCV seroprevalence reported in our study is associated with the immunocompromised status of the patients.

Despite the large sample size, this study has some limitations: firstly, we only used ELISA test for HCV screening without HCV RNA test, thus, we were unable to identify patients with active HCV infection. This could therefore lead to an over-estimation of HCV prevalence. However, previous studies suggested a high positive predictive value for the ELISA test when used alone (31). Secondly, the cross-sectional design of this study cannot establish cause and effect relationship between HCV infection and T2DM. Hence, we need to perform well designed longitudinal studies to establish the causal relationship between these two diseases in our setting. Thirdly, we did not assess liver function (score of fibrosis, liver enzyme) of HCV infected patients with type 2 diabetes mellitus, so we were unable to know the stage of HCV infection.

## 5 Conclusion

The seroprevalence of HCV infection is significantly higher among Cameroonian adults with type 2 diabetes mellitus patients than in non-diabetic controls, and is associated with increased age. This age-dependent association may suggest a generational exposure that may no longer exist overtime.

## Data availability statement

The raw data supporting the conclusions of this article will be made available by the authors, without undue reservation.

## Ethics statement

The studies involving human participants were reviewed and approved by the National Ethics Committee for Human Health Research at the Ministry of Public Health, Cameroon (Ethical clearance N°2014/04/437/I/CNERSH/SP). The patients/participants provided their written informed consent to participate in this study.

## Author contributions

ES and SPC conceived the study and designed the protocol. CF, MPK, AOB, JCK, MG-F, SZ and MYD were involved in data collection and data analysis. ES, SPC, MPK and MYD interpreted data. CF, MG-F and AOB wrote the first draft of the manuscript. ES, SPC, CF, MPK, AOB, JCK, MG-F, SZ and MYD critically revised successive drafts of the paper. ES and SPC approved its final version. ES supervised the overall work and is the guarantor of the study. All authors contributed to the article and approved the submitted version.
